# Acute Encephalopathy Caused by Inherited Metabolic Diseases

**DOI:** 10.3390/jcm12113797

**Published:** 2023-05-31

**Authors:** Yohei Sugiyama, Kei Murayama

**Affiliations:** 1 Department of Metabolism, Chiba Children’s Hospital, Chiba 266-0007, Japan; y.sugiyama.nc@juntendo.ac.jp; 2 Department of Pediatrics, Faculty of Medicine, Juntendo University, Tokyo 113-8431, Japan; 3 Center for Medical Genetics, Chiba Children’s Hospital, Chiba 266-0007, Japan; 4 Diagnostics and Therapeutics of Intractable Diseases, Intractable Disease Research Center, Graduate School of Medicine, Juntendo University, Tokyo 113-8431, Japan

**Keywords:** acute encephalopathy, inherited metabolic disease, urea cycle disorders, amino acid metabolism disorders, fatty acid metabolism disorders, mitochondrial diseases, homocystinuria, liver transplantation, enoyl-CoA hydratase short-chain 1, *THTR2* (*SLC19A3*) gene mutation

## Abstract

Acute encephalopathy is a critical medical condition that typically affects previously healthy children and young adults and often results in death or severe neurological sequelae. Inherited metabolic diseases that can cause acute encephalopathy include urea cycle disorders, amino acid metabolism disorders, organic acid metabolism disorders, fatty acid metabolism disorders, mutations in the thiamine-transporter gene, and mitochondrial diseases. Although each inherited metabolic disease is rare, its overall incidence is reported as 1 in 800–2500 patients. This narrative review presents the common inherited metabolic diseases that cause acute encephalopathy. Since diagnosing inherited metabolic diseases requires specific testing, early metabolic/metanolic screening tests are required when an inherited metabolic disease is suspected. We also describe the symptoms and history associated with suspected inherited metabolic diseases, the various tests that should be conducted in case of suspicion, and treatment according to the disease group. Recent advancements made in the understanding of some of the inherited metabolic diseases that cause acute encephalopathy are also highlighted. Acute encephalopathy due to inherited metabolic diseases can have numerous different causes, and recognition of the possibility of an inherited metabolic disease as early as possible, obtaining appropriate specimens, and proceeding with testing and treatment in parallel are crucial in the management of these diseases.

## 1. Introduction

Regardless of the cause, acute encephalopathy is a medical emergency that typically affects previously healthy children and young adults and often results in severe neurological sequelae or death [1,2]. In the treatment of acute encephalopathy, it is important to distinguish encephalopathy caused by inherited metabolic diseases in children and young adults. Inherited metabolic diseases that can cause acute encephalopathy include urea cycle disorders, amino acid metabolism disorders, organic acid metabolism disorders, fatty acid metabolism disorders (FAODs), mutations in the thiamine transporter gene, and mitochondrial diseases.

When inherited metabolic diseases are suspected, it is imperative to promptly perform metabolic screening tests because diagnosis requires specific tests. Proper diagnosis and treatment ultimately determine a patient’s prognosis.

## 2. Methods

The PubMed database and Google Scholar were searched for papers published through 25 December 2022, using a combination of search terms for acute encephalopathy, congenital metabolic diseases, inherited metabolic diseases, and hereditary metabolic diseases, as well as relevant references in the papers. We have also added references that were deemed important.

## 3. Prevalence of Inherited Metabolic Diseases

Inherited metabolic diseases are caused by genetic mutations that result in quantitative or qualitative abnormalities in enzymes or coenzymes, leading to the accumulation of substances that should be metabolized or a deficiency of substances essential for certain metabolic pathways. Each inherited metabolic disease is a rare disorder, but its overall incidence has been reported to be 1 in 800–2500 patients [3].

## 4. Diagnosis When an Inherited Metabolic Disease Is Clinically Suspected (Figure 1)

Clinical signs and symptoms of deterioration of consciousness that occur as a result of inherited metabolic diseases [1,4]:

- Often occur with little warning in a previously healthy infant or child;
- Could be missed because the early signs may be mistaken as a behavior disorder;
- Often progress rapidly, and may fluctuate markedly;
- Usually show no focal neurologic deficits;
- Can result in encephalopathy, which can be triggered by fever.

In addition to acute encephalopathy, the following symptoms indicate an underlying inherited metabolic disease [5]:

a. Sudden deterioration of general condition after infection or fasting;
b. Specific facial and skin findings, body odor, and urine odor;
c. Hypercapnia and/or respiratory disturbances associated with metabolic acidosis;
d. Growth retardation and/or mental retardation;
e. Cardiomyopathy;
f. Hepatosplenomegaly (hepatomegaly without splenomegaly, splenomegaly without findings of portal hypertension);
g. Presence of poorly related multisystemic symptoms;
h. Specific imaging findings;
i. Family history of inherited metabolic diseases.

**Figure 1:**
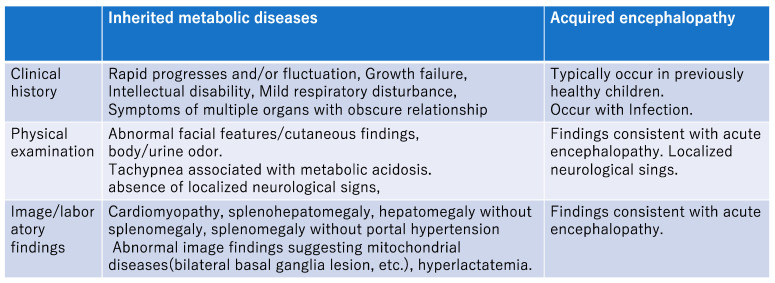
Differentiation of acute encephalopathy due to inherited metabolic diseases and acquired encephalopathies.

## 5. Typical Inherited Metabolic Diseases That Cause Acute Encephalopathy (Figure 2) 

There are various causes of acute encephalopathy. We describe main causes of acute encephalopathy.

**Figure 2:**
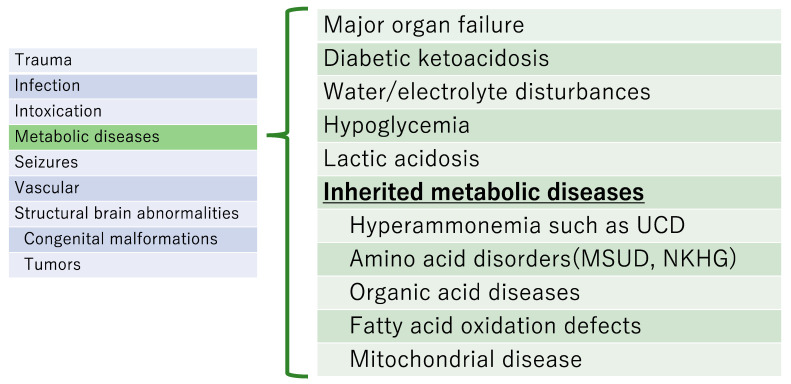
Summary of major causes of acute encephalopathy. UCD, urea cycle disorder; MSUD: Maple syrup urine disease; NKHG, Non-ketotic hyperglycinemia.

### 5.1. Urea Cycle Disorders 

Urea cycle disorders present with hyperammonemia due to genetic dysfunction of an enzyme component of the urea cycle [6]. A urea cycle disorder is strongly suspected when serum ammonia is elevated, anion gap is normal, hypoglycemia is absent, and blood urea nitrogen is decreased. Some urea cycle disorders are outlined below.

Ornithine transcarbamylase (OTC) deficiency is an X-chromosome-linked trait. Severe hyperammonemia in the neonatal period is common in males and often results in death. However, mild forms have also been observed. The clinical presentation of female symptomatic carriers varies, even when present in the same family, depending on the pattern of X-chromosome inactivation, also called lyonization, in the liver. Late-childhood presentations may include poor food intake, growth retardation, intermittent gait disturbances, and encephalopathy. Patients who were previously healthy may rapidly develop hyperammonemia and require immediate surgery (i.e., liver transplantation), but often die shortly thereafter.

Citrullinemia (classic type) often presents as hyperammonemia during the neonatal period. The presence of citrulline in blood amino acid analysis distinguishes it from OTC deficiency, which produces urinary orotic acid. 

Citrin deficiency reportedly occurs in two forms: neonatal intrahepatic cholestasis caused by citrin deficiency, which develops during the neonatal period and infancy, and adult-onset citrullinemia type 2, which develops in adulthood. During the neonatal period, the symptoms include jaundice, cholestasis, and liver damage, which may need to be distinguished from biliary atresia. In adults, acute hyperammonemia, acute encephalopathy, disorientation, delirium, and coma may occur following infection or drug or alcohol ingestion; in such cases, death may occur rapidly.

### 5.2. Amino Acid Metabolism Disorders

Maple syrup urine disease (MSUD) (leucine encephalopathy) [7] is a genetic disorder caused by a genetic deficiency in the branched-chain alpha-keto acid dehydrogenase (BCKDH), which catalyzes the decarboxylation of alpha-keto acids derived from the metabolism of branched-chain amino acids (BCAA). It may present as acute encephalopathy in the neonatal period and occur before the results of neonatal mass screening become available.

There are several types of MSUD, from the “classic type”, in which severe central nervous system (CNS) symptoms with ketoacidosis occur in the neonatal period, to the “intermittent type”, which is usually asymptomatic and shows no abnormalities on examination, but can become acutely exacerbated by infections or other events. The main treatment of MSUD is BCAA restriction using BCAA milk and food.

### 5.3. Fatty Acid Metabolism Disorders 

Acute encephalopathy due to FAODs often develops as Reye’s syndrome or a Reye-like syndrome. In FAODs, the loss of β-oxidation enzyme function results in an energy deficit because the physiological energy backup system stops functioning. Although the general laboratory and physical findings are normal, the onset of FAODs is often fatal [4]. In FAODs, severe hypoglycemia, rhabdomyolysis, Reye’s syndrome, and a Reye-like syndrome typically occur when the energy demand increases and/or energy supply decreases, leading to diarrhea and vomiting. Short-chain acyl CoA dehydrogenase deficiency is rare but presents as encephalopathy with metabolic acidosis in the neonatal period.

Although newborn mass screening with tandem mass spectrometry has been conducted nationwide since 2014 in Japan, it should be noted that not all cases are diagnosed, even if the disease is included in the screening list. Carnitine palmitoyltransferase (CPT) 2 deficiency was recognized as a cause of sudden infant death syndrome and was added to the primary category of newborn mass screening in 2017.

### 5.4. Mitochondrial Disease [8,9,10]

Mitochondrial disease is an inborn error of metabolism with a variety of organ manifestations, primarily caused by disorders of the respiratory chain or oxidative phosphorylation, and has a prevalence of 1 in 5000 patients. It often presents with characteristic imaging findings.

Mitochondrial diseases that cause acute encephalopathy are often clinically diagnosed as Leigh encephalopathy; a Reye-like syndrome; or mitochondrial myopathy, encephalopathy, lactic acidosis, and stroke-like episodes (MELAS). CNS symptoms include seizures, myoclonus, ataxia, stroke-like symptoms, decreased intelligence, migraines, psychiatric symptoms, dystonia, and myelopathy. Mitochondria are present in all cells and can damage the tissues and organs. As the name implies, MELAS is a disorder caused by abnormal mitochondrial function. Mitochondrial dysfunction causes repeated stroke-like seizures, primarily in the muscles and brain. Brain lesions often occur in the temporal and occipital lobes, and visual symptoms such as homonymous hemianopsia and cortical blindness are common. Most cases are maternally inherited, and the clinical picture is highly heterogeneous. Brain magnetic resonance imaging (MRI) shows edematous lesions with cortical predominance, which may explain why the symptoms in vascular areas are different from those usually involved in cerebral infarction. The locations of the lesions were characterized by temporal and spatial changes. Leigh syndrome is clinically diagnosed based on typical clinical manifestations and characteristic computed tomography (CT) or MRI findings. Brain imaging studies of Leigh encephalopathy have shown low-absorption areas in the basal ganglia and brainstem on brain CT and low-attenuation areas in the brain. Leigh encephalopathy has pathological features such as symmetrical spongiform changes in the brainstem and basal ganglia, necrotic changes with capillary hyperplasia, and gliosis. Several studies have comprehensively evaluated clinical genotype–phenotype correlations in extensive case series [11]. It is important to identify the biochemical and genetic backgrounds of the disease in each case because they contribute to the overall prognosis.

**Figure 3:**
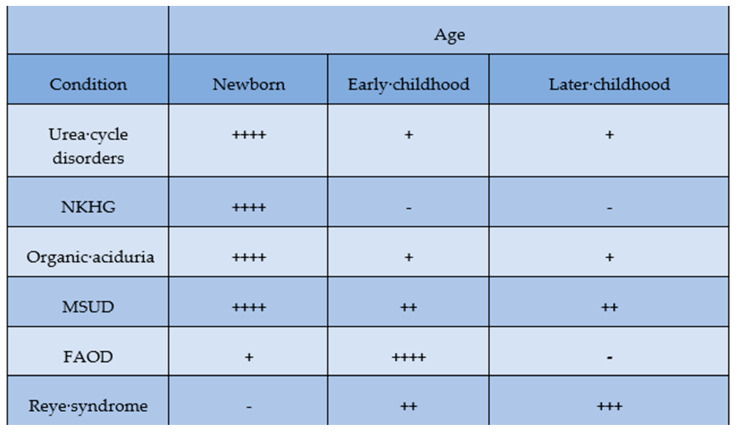
Causes of metabolic acute encephalopathy to be considered at various ages [2]. Abbreviations: NKHG: nonketotic hyperglycinemia; MSUD: maple syrup urine disease; FAOD: fatty acid oxidation disorders; -: almost none, +: sometime, ++: often, +++: frequently, ++++: most frequently.

Causes of metabolic acute encephalopathy vary according to age (Figure 3). In the neonatal period, hypoxic-ischemic encephalopathy and nuclear jaundice are specifically associated with acute encephalopathy. This should be recognized because there are specific treatments for each disease, which are hypothermia therapy and phototherapy. 

## 6. Examinations to Perform When Inherited Metabolic Diseases Are Suspected

**Figure 4:**
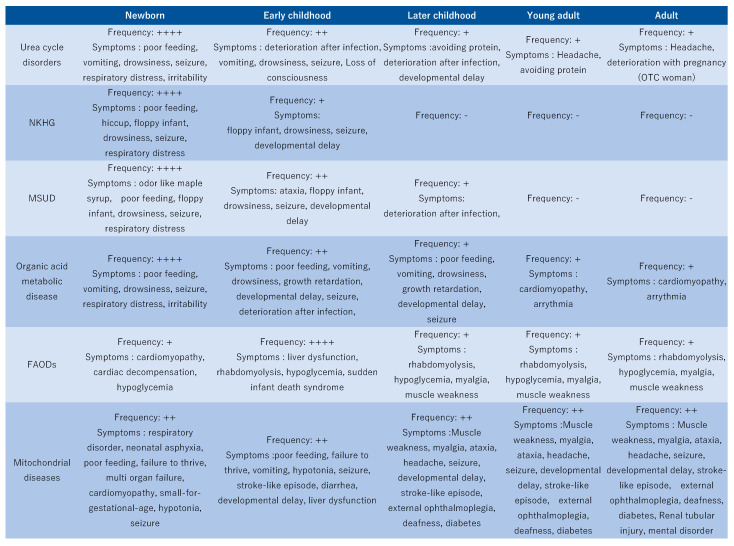
Frequency and possible symptoms of inherited metabolic diseases to be considered at various ages. OTC: Ornithine transcarbamylase deficiency. -: almost none, +: sometime, ++: often, ++++: most frequently.

If inherited metabolic diseases are suspected by symptoms(Figure 4), first-line screening tests or examinations should be performed. These include testing for serum levels of glucose, ammonia, lactic acid, pyruvic acid, blood ketones, urinary ketones, free fatty acids, blood thiamine, and homocysteine and testing for arterial blood gas levels [12]. Lactic acid, pyruvic acid, serum ketones, and free fatty acids were measured simultaneously. Ketones are measured as acetoacetate in the urine and 3-hydroxybutyrate in the serum; the latter is important because it may diverge into pathological conditions. At the very least, urinary ketones must be measured. The differentiation of each disease is shown in Table 1.

If any of these results are abnormal, clinicians should suspect inherited metabolic diseases and immediately perform further tests and examinations. Even if the results are not immediately available, a critical sample should be obtained before treatment. A critical sample is often used to confirm the diagnosis. Second-line examinations include serum amino acid, urinary organic acid, and acylcarnitine analyses (tandem mass spectrometry) using filter paper serum.

### 6.1. Laboratory Tests Specific for Each Disease

#### 6.1.1. Urea Cycle Disorders 

Serum and urine amino acid and urinary organic acid analyses are necessary to diagnose urea cycle disorders [6]. Therefore, amino acid analysis should be performed as early as possible. The results of this test will help to determine the diagnosis and treatment, particularly the dosage of arginine:

(1) Amino acid analysis in blood and urine

Abnormally high or low levels of specific amino acids, such as citrulline, argininosuccinic acid, arginine, lysine, cystine, or ornithine, may indicate citrullinemia type I, argininosuccinuria, argininemia, or hyperornithinemia-hyperammonemia-homocitrullinuria syndrome, which may be almost completely diagnosed based on the aforementioned results. Low citrulline levels are crucial for the diagnosis of carbamoyl phosphate synthetase 1 (CPS1) deficiency, N-acetylglutamate synthetase deficiency, and OTC deficiency;

(2) Urine organic acid analysis

Urine organic acid analysis is particularly important when urea cycle disorders are suspected, especially when urinary orotic acid is involved. Measurement of orotic acid is useful in the diagnosis of OTC deficiency in female patients or carriers. An allopurinol challenge test increases urinary orotate excretion; however, false negative results are common;

(3) Enzyme diagnosis or genetic analysis

Genetic diagnosis is covered by insurance and is useful for urea cycle disorders. For specific enzyme diagnosis, it is necessary for the laboratory to measure the activity of each enzyme;

(4) Tandem mass test

An increase in citrulline levels is observed in patients with citrullinemia type I and argininosuccinuria. An increase in arginine levels is observed in argininemia; however, this disease is generally excluded.

#### 6.1.2. Amino Acid Metabolism Disorders

##### MSUD (Leucine Encephalopathy)

The diagnosis of MSUD requires a prompt serum amino acid analysis. General examination reveals ketoacidosis, but patients with the intermittent forms are often missed.

The diagnosis is almost always confirmed by the presence of a peculiar urine odor that resembles the smell of maple syrup during acute exacerbation, an increase in BCAAs in serum amino acid analysis, a decrease in alanine, the appearance of alloisoleucine, and a large amount of BCKA detected in urine organic acid analysis [13]. In addition, BCKA dehydrogenase complex activity in lymphocytes and skin fibroblasts should be measured. Genetic analysis is not used for diagnosis in Japan because no mutations specific to Japanese individuals have been found.

#### 6.1.3. Fatty Acid Metabolism Disorders

Non-ketotic or hypoketotic hypoglycemia is the most characteristic feature of FAODs [14]. FAODs must be suspected when ketone levels are lower than expected, based on the degree of hypoglycemia and the patient’s general condition. We should simultaneously measure the serum total ketone body fraction (TKB) and serum free fatty acids (FFA) during hypoglycemia. If the FFA/TKB molar ratio is greater than 2.5 and the FFA/3-hydroxybutyrate molar ratio is greater than 3.0, a fatty acid metabolism disorder should be suspected.

The main tests that should be performed are as follows:

(1) Acylcarnitine analysis

Use filter paper serum. Serum acylcarnitine analysis should be performed, particularly when CPT2 deficiency is suspected. Filter paper serum should be stored at −20 °C after drying, as the test results will change if left at room temperature. Tandem mass findings in the intermittent phase may be poor and unreliable;

(2) Urine organic acid analysis

Non-hypoketotic dicarboxylic aciduria is a condition in which the patient has a hypoglycemic attack but dicarboxylic acid, a fatty acid metabolite, is absent. The intermittent phase of hypoglycemia is often considered to have no clinical findings;

(3) Enzymatic diagnosis

Enzymatic activity using peripheral blood lymphocytes or cultured skin fibroblasts can be performed;

(4) In vitro probe assay (evaluation of β-oxidation capacity)

Cultured lymphocytes and skin fibroblasts are used to indirectly evaluate the fatty acid metabolic capacity;

(5) Immunoblotting

The diagnosis is made using antibodies against enzymes to detect protein loss or by a clear decrease in the amount of protein;

(6) Genetic analysis

Genetic analysis is performed for all FAODs.

#### 6.1.4. Mitochondrial Disease [15,16,17,18]

Mitochondrial diseases are comprehensively diagnosed through clinical, imaging, and biochemical test findings (e.g., results from certain enzyme activities, pathological tests, and genetic tests):

(1) Clinical findings

Symptoms can occur in all tissues and organs, but are suspected when accompanied by multiple organ dysfunction that is poorly related to the disease (e.g., deafness, cardiomyopathy);

(2) Imaging findings

Typically, Leigh encephalopathy is observed as a high-signal area on bilateral symmetric T2-weighted and FLAIR images of the basal ganglia and brainstem on head MRI. MELAS presents with edematous lesions, predominantly in the cerebral cortex, when the symptoms appear;

(3) Hyperlactatemia

Hyperlactatemia is often present; however, lactate levels may be normal. Typically, the lactate-to-pyruvate (L/P) ratio will be greater than 20. In pyruvate dehydrogenase complex deficiency, the L/P ratio is not elevated;

(4) Enzyme analysis

Enzyme analysis is highly specific for the diagnosis of mitochondrial diseases. Enzyme activities are measured in diseased tissues or cultured cells to determine their activity in relation to the electron transfer system, pyruvate metabolism, citric acid circuit, and lipid metabolism;

(5) Pathological examination

Pathological examinations are highly specific for the diagnosis of mitochondrial diseases. Reduced activity or red-ragged fibers in skeletal muscle pathology, vessels with high succinate dehydrogenase activity, and cytochrome c oxidase-deficient fibers are the findings. Abnormal mitochondrial pathology findings are observed by electron microscopy;

(6) Genetic analysis

Abnormalities in nuclear and mitochondrial genes are the most common causes, and nuclear gene abnormalities are more common in children. However, the genotype does not correlate well with the clinical type; therefore, evaluation of enzyme defects and metabolites based on genetic abnormalities may be necessary. In some cases, a specific type of enzyme deficiency, along with its associated genetic variants and mutation rates, must be noted.

## 7. Treatment for Inherited Metabolic Diseases

If the laboratory or test results obtained at the time of initial diagnosis raise the suspicion of a congenital metabolic disease, treatment should be initiated simultaneously with a second-line examination, mostly depending on the patient’s condition and situation. In all cases, hypoglycemia must be corrected and intravenous glucose should be administered as needed. Ultimately, a glucose infusion rate of 8–10 mg/kg/min may be required. Prolonged hypoglycemia may occur with hypocarnitinemia, even in the absence of glycogen storage disease or other diseases. Hypoglycemia may also become apparent after treatment initiation, and acute attacks on dysmetabolism are often accompanied by hypercatabolism. Therefore, it is essential to maintain blood glucose within the normal range and provide adequate caloric supplementation, mainly glucose, to prevent hypercatabolism.

Although basic, the first step in any disease is to stabilize the patient’s ABC (Airway, Breathing, Circulation) in an emergency setting. Herein, we describe several conditions in which inherited metabolic diseases may be suspected.

### 7.1. When Urea Cycle Disorders Must Be Suspected

In most cases of hyperammonemia without severe metabolic acidosis (ammonia level > 400 µg/dL [>220 µmol/L]), the following actions should be performed:

(1) Check ammonia level

If the blood ammonia level is high, tests should be performed more frequently;

(2) Blood glucose control

After correcting with glucose, a 10% glucose concentration should be achieved by combining infusions to prevent catabolism. No specific type of infusion is recommended. The target blood glucose level should be 120–200 mg/dL (6.6–11 mmol/L), and hyperglycemia should be avoided with continuous administration of rapid-acting insulin. If the blood lactate level exceeds 45 mg/dL (5 mmol/L) despite insulin administration, the glycolytic system has already stopped and the glucose concentration should be lowered [19];

(3) Protection of central nervous system

Use mannitol for cerebral edema. Since glycerol use increases NADH levels, it should also be avoided in metabolic emergencies in addition to citrin deficiency;

(4) Administration of carglumic acid (Carbaglu^®^)

Carboglutinic acid (Carbaglu^®^) should be administered for undiagnosed severe hyperammonemia even when the cause is unknown because it activates CPS1, which has been reported to be effective even in CPS1 deficiency when residual enzyme activity is present [20,21]. We have encountered several cases in which initial administration was extremely effective in other urea cycle disorders and organic acid metabolism disorders;

(5) Administration of sodium phenylbutyrate (Buphenyl^®^), sodium benzoate, and L-arginine

Sodium benzoate should be prepared and administered intravenously, if possible. As L-arginine is covered by insurance, in cases of emergency, similar l-arginine preparations using the loading test can be used;

(6) Blood purification therapy

If the blood ammonia level is elevated from the beginning or if the patient responds poorly to therapy, urgent blood purification therapy should be performed. A maximum blood ammonia level above 600 µg/dL (360 µmol/L) affects neuropsychiatric development [22] and requires immediate blood purification therapy;

(7) Nutritional management

First, the administration of all proteins should be ceased. To prevent further catabolism, a caloric intake of at least 80 kcal/kg/day should be achieved. Fat emulsion should also be used. MCT milk and oil should be administered early. Protein-free milk should also be used to meet caloric needs. Essential amino acids should be introduced early (within 24–36 h) because depletion of essential amino acids and elevated ammonia levels associated with the collapse of body proteins may occur if essential amino acids are not administered for 72 h or longer. Hyperammonemia is not exacerbated by administration of 0.5 g/kg/day of amino acids;

(8) Administration of vitamins

Even in the absence of metabolic acidosis, vitamin administration is recommended to protect against secondary mitochondrial damage [23,24].

### 7.2. When Organic Acid Metabolic Diseases or Fatty Acid Metabolic Diseases Are Suspected [12]

In metabolic acidosis with an increased anion gap (pH < 7.2), an abnormal organic acid metabolism or fatty acid metabolism is suspected. Since the same tests are performed for circulatory and respiratory failure, stabilization of the patient’s general condition is necessary. Metabolic acidosis caused by inherited metabolic diseases cannot be easily corrected:

(1) First, stabilization of the patient’s ABC will be needed;
(2) Blood glucose control

Hypoglycemia should be corrected, while avoiding hyperglycemia;

(3) Administration of alkalizing agents

If pH < 7.2 is maintained despite improvement of circulatory and respiratory failure, administer sodium bicarbonate (HCO_3_-833 mEq/L) or trometamol (THAM infusion set^®^). The target values are pH > 7.2, pCO_2_ > 20 mmHg, and HCO_3_- > 10 mEq/L. Once the values have been corrected, the therapy must be discontinued immediately;

(4) Hemodialysis (continuous hemodialysis [CHD] or continuous hemodiafiltration dialysis [CHDF])

In the case of the dysmetabolism of organic acids, acidosis often does not improve even after 2–3 h of treatment, as described in points (1) to (3) above. If ammonia levels do not decrease despite the above-mentioned treatment, urgent hemodiafiltration therapy is necessary [19,25]. If necessary, transportation to a facility capable of rapid hemodiafiltration therapy should be considered. Continuous hemodialysis without hemofiltration is more common. If hyperammonemia is present, drug therapy similar to that used for urea cycle disorders should be initiated;

(5) Nutritional management

Nutritional management should be initiated as in the case of urea cycle disorders. If abnormalities in fatty acid metabolism are ruled out, lipid administration should be initiated. If enteral administration is possible, the MCT milk or oil should be administered early. Since excessive lipid administration may cause CK elevation in some FAODs, it is necessary to monitor the patient’s general condition after the intervention;

(6) Administration of vitamins

From the initial infusion, a vitamin cocktail should be administered. In particular, vitamin B12 (hydroxocobalamin in this case) for methylmalonic acidemia and biotin for complex carboxylase deficiency are crucial for treatment. Even in cases of neonatal tandem mass screening that may go undetected, all of the available vitamins should be administered. In particular, large doses of L-carnitine (100 mg/kg) are essential in cases with suspected organic acid metabolism abnormalities.

### 7.3. When Mitochondrial Disease Is Suspected [4]

Although hypoglycemia should be avoided in mitochondrial diseases, an overdose of glucose can lead to the overproduction of NADH in the cytosol and mitochondria, which can exacerbate the condition. Because this differs from other treatment strategies, it is important to ensure that second-line testing is performed to rule out other diseases.

(1) First, stabilization of the patient’s ABC;
(2) Blood glucose control

The target blood glucose level should be 120–200 mg/dL, and care should be taken not to administer more glucose than necessary;

(3) Administration of alkalizing agents

This process is similar to that described in organic acid metabolism disorders. Although dichloroacetic acid can improve hyperlactatemia, it is rarely used because of its side effects of peripheral neuropathy and inability to improve mitochondrial disease;

(4) Blood purification therapy

If hyperlactatemia and metabolic acidosis do not improve with the use of high doses of parenteral thiamine as a potential beneficial treatment [26], blood purification therapy should be performed. Although blood purification therapy is a symptomatic treatment, its aggressive introduction in cases of uncontrolled lactic acidosis often results in an improvement in the patient’s general condition and subsequent improvement in lactate levels.

(5) Vitamin cocktail

This cocktail contains vitamin cofactors and antioxidants necessary for mitochondrial metabolism and should be administered as early as when congenital metabolic abnormalities are suspected;

(6) Nutritional management

In nutritional management of mitochondrial disease, it is recommended that priority be given to lipids, with a calorie ratio of protein:lipid:carbohydrate = 10–15%:50%:35–40%, and 1–2 g/kg/day of lipids. Preferential and prompt intravenous administration of lipids is recommended even during the acute phase of the disease.

Specific treatment of MELAS includes:

(1) Taurine treatment for m.3243A>G mutation (tRNA modification disorder) [27]

Patients with MELAS with m.3243A>G have symptoms due to impaired taurine modification of mitochondrial tRNA. High doses of taurine promote tRNA maturation by stimulating the impaired taurine modifications. The efficacy and safety of high-dose taurine replacement therapy for the prevention of recurrent stroke-like attacks in patients with MELAS have been demonstrated;

(2) Arginine treatment for stroke prevention in MELAS [28]

Arginine therapy is used to treat vascular endothelial damage, which is believed to cause MELAS strokes. This treatment reduces the number of strokes. Systematic administration of oral and intravenous L-arginine may be therapeutically beneficial and clinically useful to patients with MELAS.

**Figure 5:**
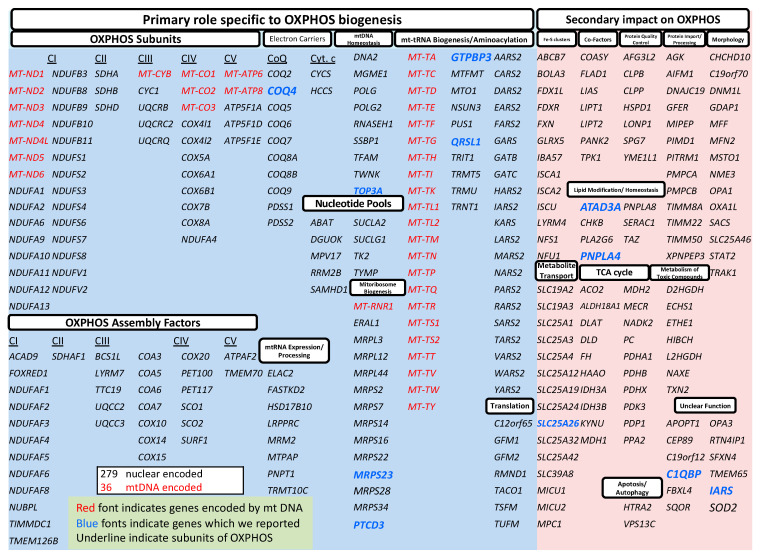
Genes linked to disorders of mitochondrial energy generation [29].

**Figure 6:**
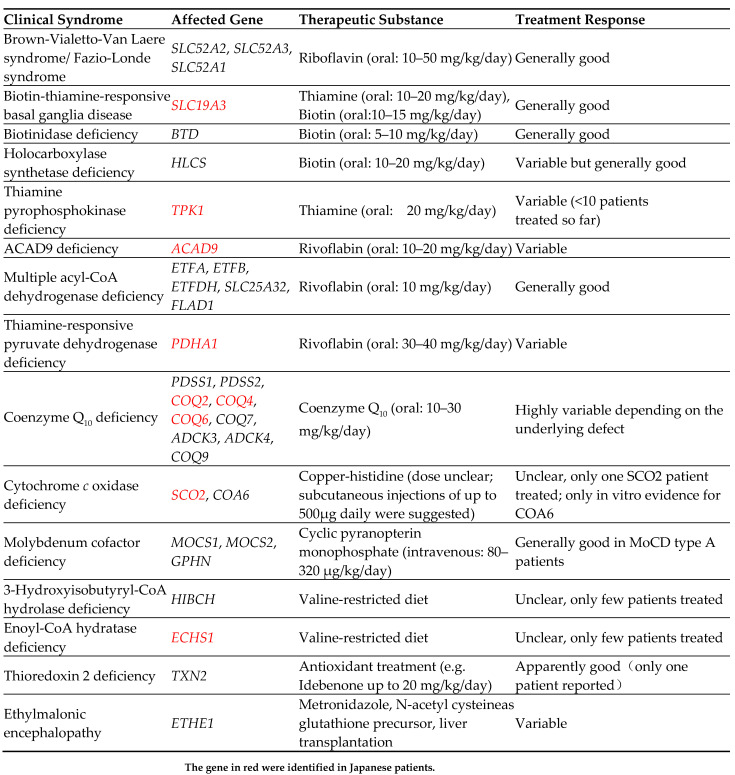
Inherited mitochondrial diseases with specific treatment options [29,30].

Mitochondrial diseases are diverse, and the processes leading to impaired ATP production differ from one gene to another, necessitating treatment (not a disease type) specific to the disease state [29] (Figure 5). Specific therapies based on the pathogenesis of mitochondrial diseases such as CoQ10 synthesis abnormalities and enoyl-CoA hydratase and short-chain 1 (ECHS-1) deficiency are emerging [30] (Figure 6). Therefore, genetic testing should be performed as soon as possible when mitochondrial disease is suspected.

## 8. Inherited Metabolic Diseases Causing Acute Encephalopathy and Other Related Disorders: Recent Findings

### 8.1. MSUD, Liver Transplantation [31]

MSUD is an inherited disorder caused by a genetic defect in the BCKDH gene, which is associated with BCAA metabolism. There are nutritional management guidelines for MSUD [32]. The goal of medical nutrition therapy in MSUD is to rapidly reduce toxic metabolites by restricting dietary BCAA to amounts that allow individuals to achieve and maintain plasma BCAA concentrations within the targeted treatment ranges. Despite progress in nutritional and medical management, classical MSUD poses a risk of serious neurological disability and untimely death. In liver, expression of BCKDH activity is 9–13% of the whole body. Liver transplantation for MSUD has been reported for around 20 years and has shown good long-term prognosis, making the lifting of postoperative protein restriction possible [33].

### 8.2. Homocystinuria Type III (5,10-Methylenetetrahydrofolate Reductase Deficiency) [34] (Figure 7), Bethain, Newborn Screening

5,10-methylenetetrahydrofolate reductase (MTHFR) deficiency is also known as homocystinuria type III. MTHFR deficiency is an autosomal recessive genetic disorder that causes hypermethioninemia and hyperhomocysteinemia owing to impaired homocysteine remethylation. Severe cases cause neurological symptoms, feeding difficulties, hydrocephalus, and death in early infancy; however, early treatment with betaine improves the prognosis. 

In a previous report, normal psychomotor development was achieved in a case of severe MTHFR deficiency in which treatment with betaine was initiated within 16 days of birth [35]. In the same report, a comparison of 19 cases in which treatment was started 17 days after birth or after the onset of symptoms revealed psychomotor retardation in almost all cases, and all 10 untreated patients died. Thrombosis may be a complication of high homocysteine levels; this is a rare but serious complication that occurs from adolescence to adulthood in this syndrome. Therefore, caution is required [36]. These reports indicate the importance of early diagnosis and treatment, not only in terms of life and neurological prognosis but also in terms of dealing with complications, especially therapeutic intervention before symptoms appear.

In some parts of Europe, patients are screened for methionine levels, methionine/phenylalanine ratio, and homocysteine levels. Pilot studies have been initiated in some areas of Japan to make the disease eligible for newborn screening.

### 8.3. ECHS-1, Valine-Restricted Diet

**Figure 8:**
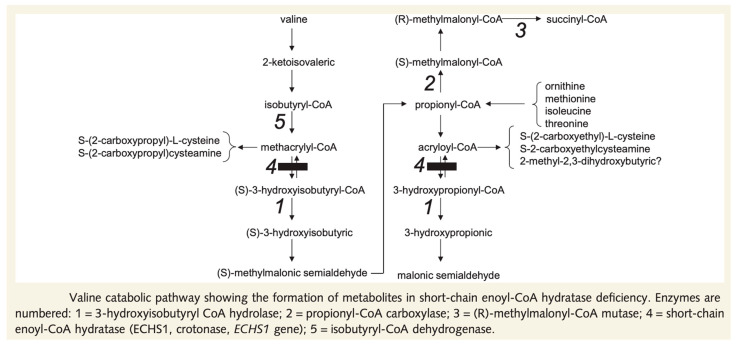
Valine catabolic pathway [38].

ECHS-1 deficiency is a mitochondrial disease that causes acute encephalopathy, and its recognition has increased in recent years. ECHS-1 deficiency is a mitochondrial disease with a broad spectrum of disease severity; however, it is often severe [37]. The symptoms of ECHS-1 deficiency include acute encephalopathy (Leigh or Leigh-like on MRI), deafness, dystonia, optic atrophy, cardiomyopathy, and lactic acidosis. ECHS-1 deficiency results in impaired amino acid metabolism, including valine and mitochondrial fatty acid beta-oxidation (Figure 8). The products of impaired valine metabolism [38], S-(2-carboxypropyl) cysteine and S-(2-carboxypropyl) cysteamine, are toxic, and the latter metabolite, 2-methyl-2,3-dihydroxybutyrate, can be identified using urine organic acid analysis and may help in diagnosis [39]. The metabolite 2-methyl-2,3-diydroxybuthyrate is very useful in screening for ECHS-1 and 3-hydroxyisobutyryl-CoA hydrolase deficiencies. Nevertheless, it can also be found in other disease groups; therefore, differentiation is necessary. Valine-restricted diets are being tested in many countries and evidence is being accumulated [40].

### 8.4. Thiamine Metabolism Dysfunction Syndrome 2 (Biotin-Thiamine-Responsive Basal Ganglia Disease), Thiamine Treatment

Thiamine metabolism dysfunction syndrome 2 (THMD2) is a genetically inherited disease reported as a treatable form of encephalopathy in Japan, Saudi Arabia, and Italy [41,42,43]. THMD2 is inherited in an autosomal-recessive manner and results from the loss of thiamine transporter 2, which is encoded by *SLC19A3* (also known as *THTR2*). The classic presentation of THMD2 occurs during early childhood and is characterized by recurrent subacute encephalopathy, although it may present in early infancy or adulthood. Treatments include the prompt administration of biotin and thiamine. Administration of biotin and thiamine early in the disease course results in partial or complete improvement within days of childhood and adulthood presentations; however, most infants with infantile presentations have poor outcomes, even after supplementation with biotin and thiamine. Biotin (5–10 mg/kg/day) and thiamine (up to 40 mg/kg/day, with a maximum of 1500 mg daily) should be administered orally as early as possible in the disease course and continued for the patient’s whole life. The thiamine dose may be doubled and administered intravenously during acute decompensation.

## 9. Conclusions

Acute encephalopathy caused by inherited metabolic diseases has various etiologies. It is important to proceed with the diagnosis in parallel with treatment. It is necessary to suspect inherited metabolic diseases as early as possible, collect critical samples, preserve them in an appropriate state, and proceed with testing.

## Figures and Tables

**Figure 7:**
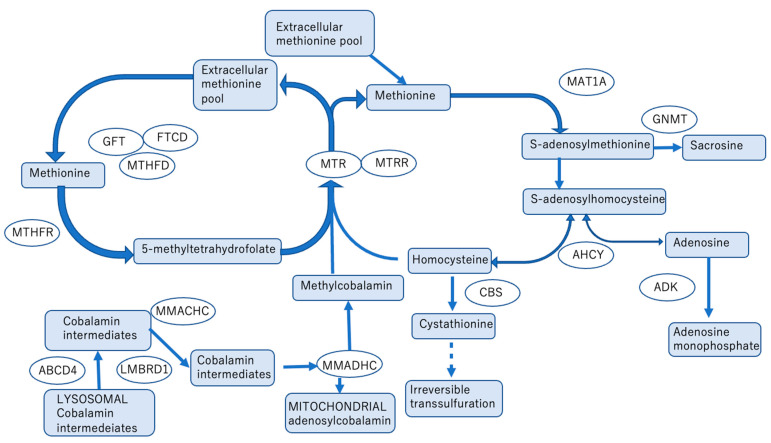
Homocystinuria conditions and methylation contributing metabolic pathways.

**Table 1:** Differentiation of inherited metabolic diseases causing acute encephalopathy.

	UCD	MSUD	OAD	FAOD	Mitochondria Disease
Metabolic acidosis	±	±	+++	±	++
Blood glucose	→	→ to ↓	↓↓	↓↓↓	→ to ↓
Ketone	→	↑↑	↑↑	↓	→
Ammonia	↑↑↑	→	↑↑	↑	→
Lactate	→	→	↑	±	↑↑↑
Liver dysfunction	→	→	→	↑↑	→
Free carnitine	→	→	↓↓↓	↓↓	→
Amino acid analysis	Specific finding	↑ BCAA	Specific finding	No finding	↑ Alanine
Urine organic acid analysis	Specific finding	Metabolites of BCAA	Specific finding	Specific finding	Specific finding

±: May or may not be present, +: present, number of + indicates the degree of changes. →: Normal, ↑: increased values are observed, ↓: decreased values are observed, number of arrows indicates the degree of changes. UCD: Urea cycle disorder, MSUD: Maple syrup urine disease, OAD: Organic acid disorder, FAOD: Fatty acid oxidation disorder, BCAA: Branched chain amino acid.

## Data Availability

Data sharing not applicable. No new data were created or analyzed in this study. Data sharing is not applicable to this article.
